# Timing and pattern of growth faltering in children up-to 18 months of age and the associated feeding practices in an urban setting of Sri Lanka

**DOI:** 10.1186/s12887-022-03265-7

**Published:** 2022-04-11

**Authors:** K. Sithamparapillai, D. Samaranayake, V. P. Wickramasinghe

**Affiliations:** 1 Professorial Paediatric Unit, Lady Ridgeway Hospital for Children, Colombo, Sri Lanka; 2 Department of Community Medicine, Faculty of Medicine, University of Colombo, Colombo, Sri Lanka; 3 Department of Paediatrics, Faculty of Medicine, University of Colombo, Colombo, Sri Lanka

**Keywords:** Growth faltering, Breastfeeding, Complementary feeding

## Abstract

**Background:**

Growth faltering is commonly encountered in breastfed infants during 4—6 months of age in low socioeconomic communities. The objective of this study was to describe the changes of growth indices with age, timing of growth faltering and its association with the feeding practices in children up-to 18 months of age.

**Methods:**

A cross sectional descriptive study was conducted in 254 children aged 12 and 18 months attending an immunization clinic. Data on growth were extracted from the Child Health Development Record. Weight and length were measured using standard methods. Feeding practices were assessed using interviewer-administered questionnaire. A drop of > 0.25 in weight-for-age Standard Deviation Score (SDS) from birth SDS was defined as weight faltering.

**Results:**

Weight faltering occurred at some point in 64.2% (*n* = 163) during first 18 months of life, and 78.5% of whom, had the onset ≤ 4 months of age. Majority (76.6%, *n* = 98) with weight for age faltering by 4 months remained so at 12 months (*p* = 0.497), while 29.7% (*n* = 38) had a weight-for-length below-2SD (*p* < 0.001). Prevalence of weight faltering was 50.4%, 46.1%, 48.4% and 48% at 4, 6, 9 and 12 months respectively. Exclusive breastfeeding was given at least until 4 months in 88% (*n* = 223) and up to 6 months in 60% (*n* = 153) while 92.9% (*n* = 236) were breastfed at 12 months, with 38.2% (*n* = 97) were breastfed on demand after six months. Complementary feeding (CF) was started before 6 months in 40.6% (*n* = 52) with early weight faltering, but only 20.3% received it with proper consistency. Breastfeeding throughout the night was significantly associated with current weight-for-length being < -1SD (OR = 1.89, CI, 1.04—3.45; *p* = 0.037).

**Conclusions:**

Early growth faltering was found in this population with high exclusive breastfeeding rates and persisting growth faltering was associated with poor feeding practices. Therefore, timely individualized interventions need to be taken to improve long term growth.

## Background

Child undernutrition is one of the main public health challenges seen in the low and middle income countries [1]. It contributes to mortality and several long term negative consequences including impaired intellectual development, low adult height and reduced economic productivity [2, 3].

Growth faltering mainly occurs during the first 2 years of life [2, 4, 5]. The WHO Global Database on Child Growth and Malnutrition which includes the surveys from developing countries shows that mean weight starts to falter at about 3 months of age which continue to decline until the catch up growth which is observed only after 18 months [2].

Optimal breastfeeding practices and timely introduction of nutritionally adequate complementary feeding are important strategies to prevent growth faltering during the first thousand days of life [6]. Health benefits of breastfeeding are well recognised beyond any doubt. In 2001 World Health Organization has recommended exclusive breastfeeding for the first six months of life [7, 8]. But, growth faltering has been described in 4–6 month old breastfed infants in less developed communities [9–11].

Maternal nutrition and diet could have effects on the breastmilk content and composition. Maternal BMI was reported to be positively correlated with breastmilk energy, fat and dry matter [12]. A systematic review by Daniel et al. revealed that although the fat content of breastmilk increased with increase in BMI of mothers, there was no significant difference in the energy content of breastmilk compared to lean mothers [13]. Micronutrient inadequacy of breast milk especially with regard to the iron content is also of concern and maternal iron deficiency leading to lower iron stores in the newborn aggravates iron deficiency in infancy [8]. A study done in Indonesian infants reported that the intake of majority of micronutrients were below the recommendations in mothers and in their exclusively breastfed babies [14].

WHO’s recommendation on the optimal duration of exclusive breastfeeding also states that the available data are insufficient to exclude several other potential risks associated with exclusive breastfeeding for six months including growth faltering and micronutrient deficiencies in some infants. Therefore, interventions should be individualised if there is any concern regarding the growth of the infant [7].

According the Demographic Health Survey—2016, in Sri Lanka 21% of children under the age of 5 years were underweight, 17% were stunted and 15% were wasted [15]. These figures are much higher, than that would be expected for a country with low levels of infant mortality and high female literacy rates, and remains unchanged over the past few decades inspite of high exclusive breastfeeding rates, regular growth monitoring and other public health interventions.

Timing, patterns and determinants of child growth differ according to country, region and cultural practices. Therefore, knowledge on these aspects in a population would help to implement specific interventions to uplift the nutritional status. This study aimed to describe the changes of growth indices with age, timing of growth faltering and its association with the feeding practices in children up-to 18 months of age.

## Methods

### Participants

A cross sectional descriptive study was conducted in 254 children aged 12 and 18 months attending an immunization clinic in a tertiary hospital in Colombo, Sri Lanka. Children who were attending for their 12 or 18 month vaccination were invited and recruited consecutively to the study until the required numbers were achieved. Children with chronic medical conditions and congenital deformities and chromosomal disorders were excluded. Children with cerebral palsy and congenital deformities which causes feeding difficulties (e.g. cleft palate) were also excluded.

### Data collection

Interviewer administered pre-tested questionnaire was used to collect data on demographic and socio-economic status, and feeding practices. Structured questions were included to collect information regarding breastfeeding, formula-feeding and complementary feeding practices.

Exclusive breastfeeding was defined as feeding / having been fed nothing other than breastmilk for nutritional or fluid providing purposes. Formula feeding is defined as feeding / having been fed commercial formula milk of non-human origin. Complementary feeding is defined as feeding any solid or semi-solid food or liquid to infants in addition to breastmilk or formula milk for nutritional or fluid providing purposes. Complementary food which is thick enough to stay on the spoon without running off when the spoon is tilted is defined as the proper consistency of CF. Images on the Child Health Development Record of Sri Lankan children showing the proper and improper consistency of CF were used during data collection.

The following socio-demographic and economic variables were collected; Mother’s age at recruitment in completed years, mother’s employment status at recruitment (housewife / employed) and mother’s highest educational qualification. Average family monthly income in Sri Lankan Rupees (LKR) was collected. Economic status was categorized into low (USD < 157), middle (USD 157—315) and high (USD > 315) (conversion at 1 USD = 146 LKR, Central Bank of Sri Lanka, 2016) according to the Sri Lankan household income and expenditure survey 2016 [16].

Weight, length and occipitofrontal circumference (OFC), at point of recruitment ie. 12 and 18 months, were measured using standard protocol by a single investigator [17]. Anthropometric measurements at birth, and at 3, 4, 5, 6, 9 and 12 months of age were extracted from the Child Health Development Record (CHDR). SDSs for weight-for-age and length-for-age at birth and at 3, 4, 5, 6, 9, 12 and 18 months were calculated using LMS growth package (WHO 2007) operating on Excel programme of Microsoft Office. Based on the significance of faltering in weight, a drop of > 0.25 SDS in weight-for-age from birth SDS was defined as weight faltering. A rise of ≥ 0.67 SDS in weight-for-age from birth SDS was defined as accelerated weight gain [18]. Nutritional statuses, underweight, stunting and wasting were assessed using weight-for-age, length-for-age and weight-for-length indices respectively at each time points.

### Analysis

The data was analysed using SPSS version 21 statistical package. Data is presented as mean (SD) and frequency distribution tables as appropriate. Categorical data was analysed using Chi square tests. The level of significance was set at a *p* < 0.05. Factors associated with weight faltering and weight-for-length were analysed using binary logistic regression.

## Results

Study population included 53.1% (*n* = 135) of 12-month-olds. There were no significant differences between 12-month and 18-month-olds in their characteristics. Table 1 shows characteristics of the study population according to the age.

**Table 1:** Characteristics of children according to the age

**Characteristics**	**Total** **(*n* = 254)**	**Age of the child**	
		**12 months** **53.1% (*n* = 135)**	**18 months** **46.9% (*n* = 119)**
Sex			
Male	141 (55.5%)	71 (50.4%)	70 (49.6%))
Female	113 (44.5%)	64 (56.6%)	49 (43.4%)
Birth Order			
First born	109 (42.9%)	63 (57.8%)	46 (42.2%)
≥ second	57.1% (*n* = 145)	72 (49.7%)	73 (50.3%)
Maturity at birth			
≥ 37 weeks	232 (91.3%)	123 (53.0%)	109 (47.0%)
< 37 weeks	22 (8.7%)	12 (54.5%)	10 (45.5%)
Birth weight			
≥ 3.5 kg	27 (10.6%)	9 (33.3%)	18 (66.7%)
2.5 – 3.49	173 (68.1%)	99 (57.2%)	74 (42.8%)
< 2.5 kg	54 (21.3%)	27 (50.0%)	27 (50.0%)
Birth interval^a^ (years)	5.2 (± 3.1)	5.02 (± 3.1)	5.4 (± 3.0)
Mother’s age			
≥ 35 years	62 (24.4%)	29 (46.8%)	33 (53.2%)
< 35 years	192 (75.6%)	106 (55.2%)	86 (44.8%)
Mother’s occupation			
Employed	56 (22%)	32 (57.1%)	24 (42.9%)
Housewife	198 (78%)	103 (52.0%)	95 (48.0%)
Mother’s education			
Up-to secondary	119 (46.9%)	68 (57.1%)	51 (42.9%)
≥ Tertiary	135 (53.1%)	67 (49.6%)	68 (50.4%)
Average monthly family income			
Low	16 (6.3%)	5 (31.3%)	11 (68.8%)
Middle	121 (47.6%)	61 (50.4%)	60 (49.6%)
High	117 (46.1%)	69 (59%)	48 (41.0%)

^a^ Birth interval is the time difference between the index and the immediate previous sibling

### Current nutritional status

When current nutritional status of the study group was assessed (in 12-month and 18-month-old children), weight-for-age SDS < -2SD was seen in 19.3% (*n* = 49) and length-for-age SDS < -2 SD in 10.2% (*n* = 26) and there was no significant difference in the two age groups when analysed separately (data not shown). Weight-for-length SDS < -2SD was seen in 17.7% (*n* = 45), and 22.2% (*n* = 10) of them were under -3SD. Current weight-for-length < -2SDwas significantly associated with low birth weight (< 2500 g) (OR = 5.5; CI, 2.33–12.97; *p* < 0.001). However, the current weight-for-length < -2SD was not significantly associated with pre-maturity-at-birth (OR = 0.26; CI, 0.068–0.99; *p* = 0.05), age of the child (OR = 1.82; CI, 0.90–3.68; *p* = 0.09), sex (OR = 1.03; CI, 0.518–2.07; *p* = 0.918) or maternal age being above 35 years (OR = 1.9; CI, 0.90–3.99; *p* = 0.09) when adjusted for maternal employment status, maternal education and family income.

### Onset and pattern of growth faltering

**Fig. 1:**
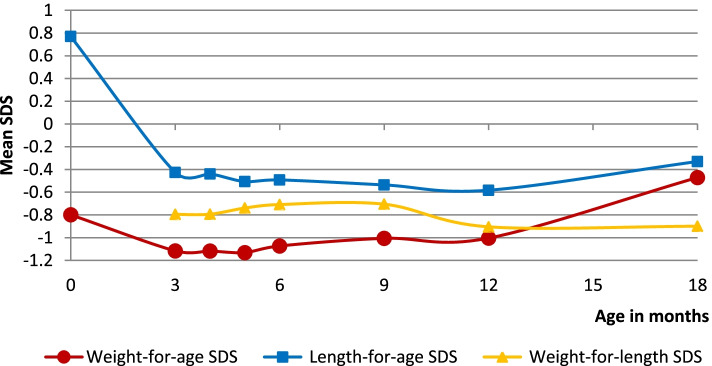
Changes in the growth parameters with age in the total population

Drops in mean weight-for-age and length-for-age SDS were noted at 4 months. Mean weight-for-age SDS dropped by 0.319 while length-for-age SDS dropped by 1.2 (Fig. 1). Weight-for-length did not show a drop between 3–9 months but dropped between 9–12 months.

Weight faltering (> 0.25 in weight-for-age SDS drop from birth weight SDS) occurred in 64.2% (*n* = 163) of children at some point during the first 18 months of life, and 78.5% (*n* = 128) of them had the onset before 4 months of age (Table 2).

**Table 2:** Age of onset of weight faltering

Age at weight faltering noted^a^	Number (%)
4 months	128 (50.4%)
6 months	6 (2.4%)
9 months	14 (5.5%)
12 months	7 (2.8%)
18 months	8 (3.1%)
Total with weight faltering	163 (64.2%)
No weight faltering	91 (35.8%)
Total	254 (100%)

^a^ The age at which weight-for-age SDS drop of > 0.25 was noted

Although the mean weight-for-age SDS showed a catch-up growth by 18 months, the prevalence of weight faltering remained high without much change until 18 months of age (Table 3).

**Table 3:** Changes in anthropometric indices with time in the total population

Changes in anthropometry	Prevalence at different ages				
	**4 months**	**6 months**	**9 months**	**12 months**	**18 months**
**Change in weight-for-age SDS compared to birth SDS**					
Weight faltered^a^	128 (50.4%)	117 (46.1%)	123 (48.4%)	122(48.1%)	63 (52.9%)
No weight faltering	85 (33.5%)	88 (34.6%)	73 (28.7%)	76 (29.9%)	32 (26.8%)
Accelerated weight gain^b^	41 (16.1%)	49 (19.3%)	58 (22.8%)	56 (22%)	24 (20.1%)
**Length-for-age SDS**					
Below -2 SD	15 (5.9%)	21 (8.3%)	20 (7.9%)	21 (8.3%)	13 (10.9%)
Between -1 to -2 SD	46 (18.1%)	48 (18.9%)	52 (20.5%)	63 (24.8%)	30 (25.2%)
**Weight-for-length SDS**					
Below -2 SD	37 (14.5%)	30 (11.8%)	27 (10.6%)	49 (20.1%)	17 (14.3%)
Between -1 to -2 SD	50 (19.7%)	56 (22%)	64 (25.2%)	68 (26.8%)	42 (35.2%)

^a^ A drop of > 0.25 in weight-for-age SDS from birth weight SDS

^b^ A rise of ≥ 0.67 in weight-for-age SDS from birth weight SDS

Weight faltering, compared to birth weight, was noted in 50.4% (*n* = 128) of infants by 4 months of age. Of them majority (76.6%, *n* = 98) remained weight faltered (*p* = 0.497) at 12 months (Table 4). Of those with weight faltering at 4 months, 29.7% (*n* = 38) had weight-for-length < -2SD (*p* < 0.001) and 16.4% (*n* = 21) had length-for-age < -2SD (*p* < 0.001) at 12 months of age.

**Table 4:** Changes in weight status with time in children with and without weight faltering occurring before 4 months of age

Weight faltering status at 4 months	Changes in weight status from 6 to 18 months				
	**Weight-for-age SDS status**	**6 months**	**9 months**	**12 months**	**18 months**
Weight faltering before 4 months (*n* = 128)	Weight faltered^a^	109 (85.2%)	102 (79.8%)	98 (76.6%)	47 (79.7%)
	No weight faltering	18 (14%)	23 (17.9%)	26 (20.3%)	8 (13.6%)
	Accelerated weight gain^b^	1 (0.8%)	3 (2.3%)	4 (3.1%)	4 (6.7%)
No weight faltering before 4 months (*n* = 126)	Weight faltered^a^	8 (6.4%)	21 (16.7%)	24 (19%)	16 (26.7%)
	No weight faltering	70 (55.5%)	50 (39.6%)	50 (39.7%)	24 (40%)
	Accelerated weight gain^b^	48 (38.1%)	55 (43.7%)	52 (41.3%)	20 (33.3%)

^a^ A drop of > 0.25 in weight-for-age SDS from birth weight SDS

^b^ A rise of ≥ 0.67 in weight-for-age SDS from birth weight SDS

Of the infants with weight-faltering at 4 months, 86.7% received exclusive breastfeeding (EBF) up to 4 months and 53.9% up-to 6 months. Complementary feeds (CF) were introduced before 6 months in 40.6% (*n* = 52) and 50% (*n* = 26) of them received it of improper consistency.

In children with weight faltering at 4 months and persistent weight faltering at 12 months (76.6%), EBF was given at least until 4 months in 88.7% and up to 6 months in 52%. Forty five percent received CF before 6 months and 31.7% was given formula feeds. Twenty three percent of children with weight faltering at 4 months, regained the original weight status at 12 months. A fourth of them (26.7%) were started on CF before 6 months, while 46.8% (*n* = 14) were given formula feeds.

**Fig. 2:**
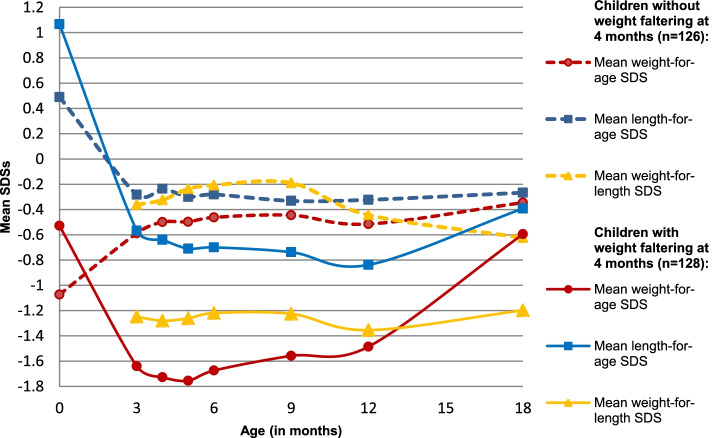
Changes in the growth parameters of children in relation to weight faltering at 4 months

Children with weight-faltering at 4 months had significantly higher mean weight-for-age SDS at birth (*p* < 0.001) and length-for-age SDS (*p* = 0.005) at birth when compared to those without weight-faltering at 4 months (Fig. 2).

Table 5 shows characteristics of the study population and the association with their overall weight faltering status.

**Table 5:** Characteristics of children according to their overall weight faltering status

**Characteristics**	**Total** **(*n* = 254)**	**Weight faltering up-to 18 months**^**a**^		OR (95% CI) significance^b^
		**Absent (*n* = 91)**	**Present (*n* = 163)**	
Sex				
Male	141 (55.5%)	52 (36.9%)	89 (63.1%)	1.00
Female	113 (44.5%)	39 (34.5%)	74 (65.5%)	1.00 (0.58- 1.71); *p* = 0.997
Birth order				
First born	109 (42.9%)	44 (40.4%)	65 (59.6%)	1.00
≥ Second	145 (57.1%)	47 (32.4%)	98 (67.6%)	0.80 (0.46- 1.4); *p* = 0.440
Birth weight (kg)				
< 2.5	54 (21.3%)	24 (44.4%)	30 (55.6%)	1.0
≥ 2.5	200 (78.7%)	67 (33.5%)	133 (66.5%)	0.35 (0.16- 0.77); *p* = 0.009
Maturity				
< 37 weeks	22 (8.7%)	5 (22.7%)	17 (77.3%)	1.0
≥ 37 weeks	232 (91.3%)	86 (37.1%)	146 (62.9%)	4.57 (1.32- 15.7); *p* = 0.016
Mother’s age				
≥ 35 years	62 (24.5%)	17 (27.4%)	45 (72.6%)	1.0
< 35 years	192 (75.5%)	74 (38.5%)	118 (61.5%)	1.57 (0.80- 3.07); *p* = 0.184
Mother’s education				
Up-to secondary	119 (46.9%)	43 (36.1%)	76 (63.9%)	1.0
≥ Tertiary	135 (53.1%)	48 (35.6%)	87 (64.4%)	1.07 (0.59- 1.94);*p* = 0.807
Mother’s employment				
Employed	56 (22%)	19 (33.9%)	37 (66.1%)	1.0
Housewife	198 (78%)	72 (36.4%)	126 (63.6%)	1.11 (0.56- 2.17);*p* = 0.755
Average family monthly income				
Low	16 (6.3%)	5 (31.3%)	11 (68.8%)	1.0
Middle	121 (47.6%)	43 (35.5%)	78 (64.5%)	0.87 (0.27- 2.78);*p* = 0.815
High	117 (46.1%)	43 (36.8%)	74(63.2%)	0.76 (0.22- 2.58);*p* = 0.669

^a^ Drop of > 0.25 in weight-for-age SDS from the birth weight SDS during the first 18 months of life

^b^ Using binary logistic regression with the presence of weight faltering as the dependant variable and sex, birth order, birth weight, maturity, mother’s age, mother’s education, mother’s employment status and average family monthly income as independent variables

### Breastfeeding practices

Ninety eight percent were ever breastfed. EBF was given for at least 4 months in 87.8% (*n* = 223) and at least 6 months in 60.2% (*n* = 153). Breastfeeding was continued in 92.9% (*n* = 236) until at least 12 months of age while 84.8% (*n* = 100) of the 18-month-old children were still breastfed.

Table 6 shows the breastfeeding practices after 6 months and its association with weight-for-length < -1SD in the total population and in those with weight faltering before 4 months. Thirty eight percent (*n* = 97) continued to breastfeed on demand after six months without considering the mealtime (i.e. not leaving adequate time interval between the breastfeed and the CF) and it was not significantly associated with weight-for-length < -1SD (OR = 1.54; CI, 0.84–2.82; *p* = 0.161) or weight-for-length < -2SD (OR = 2.01; CI, 0.91- 4.44; *p* = 0.08). Breastfeeds were continued to be given on demand after six months in 38.3% (*n* = 49) of children with weight faltering at 4 months. In this subsample on demand breastfeeding during daytime was not significantly associated with weight-for-length < -1SD (OR = 1.94; CI, 0.77–4.88; *p* = 0.157) or weight-for-length < -2SD (OR = 2.09; CI, 0.75 -5.87; *p* = 0.158).

**Table 6:** Association between breastfeeding practices after 6 months and current weight-for-length status (< -1SD) in the total population and in those with weight faltering before 4 months

BF practices	Total population (*n* = 254)			
	**Total** **(*n* = 254)**	**Weight-for-length < -1SD**^**a**^	**Weight-for-length ≥ -1SD**	OR (95% CI) significance^b^
**BF practices after 6 months during daytime**				
Continued on-demand BF	97 (38.2%)	53 (54.6%)	44 (45.4%)	1.0
Not given on-demand BF	157 (61.8%)	68 (43.3%)	89 (56.7%)	1.54 (0.84- 2.82) *p* = 0.161
**BF practices after 6 months during night**				
Breastfed throughout the night	140 (55.1%)	76 (54.3%)	64 (45.7%)	1.0
Not breastfed throughout the night	114 (44.9%)	45 (39.5%)	69 (60.5%)	1.89 (1.04- 3.45) *p* = 0.037
	**Weight faltered before 4 months (*n* = 128)**			
**BF practices after 6 months during night**				
Continued on-demand BF	49 (38.2%)	33 (67.3%)	16 (32.7%)	1.0
Not given on-demand BF	79 (61.8%)	44 (55.7%)	35 (44.3%)	1.94 (0.77- 4.88) *p* = 0.157
**BF practices after 6 months during night**				
Breastfed throughout the night	67 (52.3%)	48 (71.6%)	19 (28.4%)	1.0
Not breastfed throughout the night	61 (47.7%)	29 (47.5%)	32 (52.5%)	2.55 (1.05- 6.2) *p* = 0.039

^a^ < -1SD cut-off was used here in order to achieve the most ideal anthropometry match

^b^ using binary logistic regression with the presence of weight-for-length < -1SD as the dependant variable and adjusted for birth weight, maturity at birth, maternal age, maternal education, employment status and family income

Breastfeeding throughout the night was practised after introduction of CF in 55.1% (*n* = 140) and it was significantly associated with current weight-for-length being < -1SD (OR = 1.89; CI, 1.04–3.45; *p* = 0.037) but did not show significant association with weight-for-length < -2SD (OR = 1.42; CI, 0.64–3.18; *p* = 0.382). Breastfeeding throughout the night after starting CF was practised in 52.3% (*n* = 67) of children with growth faltering at 4 months and was significantly associated with current weight-for-length < -1SD (OR = 2.55; CI, 1.05–6.2; *p* = 0.03). But it was not significantly associated with current weight-for-length < -2SD (OR = 1.5; CI, 0.57–3.98; *p* = 0.407).

### Complementary feeding practices

CF were introduced under 6 months in 34.3% (*n* = 87) of the total population and only in 20.1% (*n* = 51) CF of proper consistency was given. Only 40.6% (*n* = 52) with early weight faltering was given CF under 6 months while the rest continued to be exclusively breastfed despite growth faltering and only 20.3% (*n* = 26) received CF made of proper consistency. Liquids other than breastmilk and formula milk (rice kanji, clear soup, water and fruit juices) were given before the completion of 6 months in 32.7% (*n* = 83) and this was significantly associated with weight faltering at 12 months (OR = 1.9; CI, 1.06–3.37; *p* = 0.029) after adjusting for birth weight, maturity-at-birth, maternal age, employment status, maternal education and family income.

### Complementary feeding introduced before 6 months

Although 34.3% (*n* = 87) was given CF before 6 months, 41.4% (*n* = 36) of them did not receive it in proper consistency until 6 months. The reasons for introducing CF before 6 months were growth faltering (43.7%, *n* = 38) and mother’s employment (21.8%, *n* = 19) while 49.4% (*n* = 43) started CF because they believed that it would be an additional nourishment.

Sixty percent (*n* = 52) of those who received CF before 6 months had weight faltering at 4 months and 84.6% (*n* = 44) of them remained to be weight faltered at 12 months. Thirty one percent (*n* = 27) of those who received CF before 6 months had weight-for-age < -2SD at 4 months and 66.7% (*n* = 18) of them remained to have weight-for-age < -2SD at 12 months.

## Discussion

Growth faltering patterns of children differ across the world and faltering patterns are different for weight and height [2, 5, 19, 20]. Previous analysis revealed that in South Asian region, weight for age SDS dropped steadily until 18 months and continued to decrease at a slower rate until 5 years. Drop in height-for-age SDS was more than weight-for-age SDS and it dropped until 24 months with no catch-up growth thereafter among children in South Asian region [2]. But our findings showed that there is a drop of mean weight-for-age SDS and length-for-age SDS detected at 4 months and catch-up growth was seen by 18 months. Drop in length-for-age SDS was higher than weight-for–age SDS.

A drop of > 0.25 in weight-for-age SDS from birth SDS was defined as weight faltering in this study, which is the early step in the development of undernutrition. Our findings revealed that there is a high prevalence of weight faltering and in the majority weight faltering was noted at 4 months. Mean weight-for-age SDS at 18 months was higher than the mean birth SDS. But, the prevalence of weight faltering at 18 months remained almost the same as at 4 months. Although majority with weight faltering at 4 months remained weight faltered at 12 months, others with initial weight faltering gained more weight and went above their weight-for-age SDS at birth while some even had accelerated growth.

Growth faltering in these children could mostly be attributed to inappropriate feeding practices, since these children were otherwise healthy. National policy on breastfeeding in Sri Lanka is exclusive breastfeeding for the first six months of life as recommended by WHO [21]. But national guidelines also state if growth faltering occurs or if mother is not available for on demand breast feeding, CF could be started at the completion of four months [21]. High prevalence of weight faltering at 4 months was found in this study inspite of high exclusive breastfeeding rates. Early growth faltering in exclusively breastfed infants were reported in other studies as well [9, 10]. Therefore, there is a need to understand the missing contributors to this early growth faltering despite detecting at 4 months but not taking corrective action. This could probably be due to the faithful adherence to set guidelines imposed on a population level, but not knowing how to apply on an individual basis when deviation occurs.

It was noted that the children in whom weight faltering was detected at 4 months, the mean weight and length at birth were higher than the infants who did not show weight faltering at 4 months. This could possibly be due to that the breastmilk alone was not adequate to support the growth of larger babies beyond 4 months. Studies have reported that there is no marked increase in milk transfer with age and there is a ‘plateau’ in milk transfer after 3 months [22].

CF should be introduced at a time when frequent and exclusive breastfeeding alone is not adequate to fulfil the nutrient requirements of the infant [3]. Although WHO recommends introducing CF at 6 months, it is emphasized that it is applied for populations rather than individuals. Therefore introduction of CF is an individual decision and it might be necessary to start CF before 6 months to fill the gap in nutrient for infants with growth faltering [23]. Our results showed that inspite of regular growth monitoring and identification of growth faltering, timely individualized interventions were lacking in many. Therefore, it would be important to closely observe these children with a better potential to grow, should not be deprived of this opportunity but closely monitor the growth and introduce CF at an earlier stage than six months at earliest point growth faltering is noted if the child is developmentally ready to receive CF.

Evidence on CF introduction between 4 -6 moths and status of the weight of the baby is inconclusive [24, 25]. Introduction of CF before 6 months of age can lead to reduction of breastmilk intake and therefore the total energy consumption might remain the same [26]. But there is evidence showing improvement of iron status of the infant with introduction of CF at 4 months [27, 28]. Furthermore, the calorie density of properly constituted CF is much higher than of breast milk and could enhance calorie intake and thus growth. The typical Sri Lankan CF contains rice, dhal, fish and vegetables with added coconut milk. Energy density of cooked rice and dhal ranges between 130 – 150 kcal/100 g and the addition of coconut milk can increase the energy density further. Therefore, if the correct proportions of these are used and appropriate quantity of CF is provided, the calorie delivered will be much higher than breastmilk. Usually the volume consumed by a six month old baby is about 100 ml thus a child could easily consume 100 g of CF.

Our results revealed that the introduction of CF before 6 months was not associated with change in weight status at 9 or 12 months. Although CF was introduced early due to growth faltering, feeds of proper consistency and variety was introduced only after the completion of 6 months. However, data regarding the quantity of CF given was not collected in our study. This denotes that the composition of CF as well as the volume fed may have not being appropriate, and also would have deprived the full intake of breast milk as well as secondarily reducing the breast milk secretion due to less suckling by the baby. Therefore it is important that if it is decided to start CF it should be done in the proper way, that is proper consistency with the correct energy density with adequate volume and frequency with adequate spacing between meals.

Data from this study also have shown that despite having good birth weight, it has not been able to sustain during infancy. Further those showed growth faltering recovered poorly. Considering weight-for-age SDS alone without interpreting it with birth parameters in babies with good birth weight might be misleading. This has resulted in lack of timely interventions despite growth faltering in many. Defining weight faltering in relation to birth parameters would help to identify weight faltering in babies with good birth weight. This would lead to early interventions resulting in quicker recovery. Delayed interventions may lead to poor recovery of growth faltering despite interventions since they settle on a lower growth trajectory. Therefore, this study shows that growth has to be addressed on an individual basis and compared with its own birth parameters rather than on a set cut-off values. Close growth monitoring would help in early intervention and correction of the growth faltering and sustain their growth potential which is more or less denoted by birth parameters. However, a word of caution is that children who were macrosomic (birth weight > 3.5 kg in Sri Lankan context), could be due to hyperglycaemia of pregnancy and would have a tendency to show physiological catch down to achieve its correct genetic potential of growth. Overlooking this and trying to correct could lead to development of overweight from a young age.

Data on maternal nutritional status was not collected in our study and it is a limitation. But maternal low BMI is not of a major concern in this population. Prevalence of low (< 18.5 kg/m^2^) and high BMI (> = 25 kg/m^2^) among mothers in the urban area was 7.2% and 44.6% respectively in 2016 [29]. But, micronutrient content of breastmilk could be different between mothers of infants experiencing growth faltering and those that did not. This could be one of the missing contributors for early growth faltering in breastfed children.

This is a cross sectional study, which also used secondary data extracted from the CHDR could be considered reliable, as it is the document that is maintained for every child from birth and carry a valid set of measurements collected by trained Public Health Midwives, and used as a screening tool where interventions would be made. But all the measurements were not taken by the same investigator and it is a limitation in the study. Feeding practices were assessed with the use of questionnaire and recall bias could be a limitation of our study. Data regarding the quantity of CF and consumption of fruits and vegetables was not collected and the dietary diversity was not calculated.

## Conclusions

The findings show the high prevalence of growth faltering in this population until 18 months of age. Onset of growth faltering is seen very early in life in a majority of infants inspite of high exclusive breastfeeding rates. However, many persisted with exclusive breast feeding without starting CF, despite detecting growth faltering at an age earlier than 6 months. Therefore, growth monitoring and timely intervention with timely initiation of proper breastfeeding and complementary feeding practices need to be strengthened to improve long term growth. This is a small cross sectional study done only in the subjects from the urban sector of Sri Lanka to identify time point of growth faltering, action taken and its impact on overall growth. Further studies are needed to confirm these findings and to find the other confounders to the growth faltering while infants are on exclusive breastfeeding. These will help to implement evidence-based guidelines for the management of early growth faltering to achieve the full potential of growth and development of infants.

## Data Availability

The datasets used and /or analysed during the current study are available from the corresponding author on reasonable request.

## Abbreviations

CHDR: Child Health Development Record
CF: Complementary feeds
EBF: Exclusive Breastfeeding
FF: Formula Feeds
SDS: Standard Deviation Score
